# Causal effects of glutamine and lipid-related metabolites on alopecia areata: A 2-sample Mendelian randomization study

**DOI:** 10.1097/MD.0000000000046312

**Published:** 2025-12-12

**Authors:** Xiao-Shuang Yang, Ru Dai, Yu-Jie Miao, Sheng-Zhao Chen, Zhong-Fa Lv

**Affiliations:** a Department of Dermatology, Second Affiliated Hospital, Zhejiang University School of Medicine, Hangzhou, Zhejiang Province, P.R. China.

**Keywords:** alopecia areata, blood metabolites, hair follicle, Mendelian randomization study

## Abstract

Alopecia areata (AA) is an autoimmune hair loss disorder that affects approximately 2% of the global population, imposing a substantial psychological burden and impairing the quality of life. Although observational studies have correlated blood metabolites with AA, these associations are susceptible to confounding and reverse causality, leaving the causal direction unclear. This study employed a 2-sample Mendelian randomization (MR) approach to infer the causal relationship between blood metabolites and AA. This study used publicly available genome-wide association study summary statistics for blood metabolites (Kettunen et al, N = 24,925) and AA (FinnGen, 682 cases/361,140 controls), both based on European ancestry populations. Single-nucleotide polymorphisms significantly associated with metabolites were selected as instrumental variables, while those in linkage disequilibrium or with an *F*-statistic < 10 were excluded to ensure the robustness of the instruments. The primary analysis utilized the inverse variance weighted method, supplemented by MR-Egger, weighted median, and MR-PRESSO analyses to assess horizontal pleiotropy and heterogeneity. A leave-one-out sensitivity analysis was performed to evaluate the influence of individual single-nucleotide polymorphisms. Forward MR analysis identified 25 blood metabolites suggesting potential causal effects on AA. Specifically, glutamine exhibited a protective effect (odds ratio [OR] = 0.59, 95% confidence interval [CI]: 0.35–0.99). Positive associations were observed for the ratio of free cholesterol to cholesterol esters (OR = 1.34, 95% CI: 1.04–1.74), serum total cholesterol (OR = 1.31, 95% CI: 1.02–1.68), citric acid (OR = 2.89, 95% CI: 1.41–5.92), and glucose (OR = 1.99, 95% CI: 1.01–3.92). Notably, lipoprotein-related metabolites accounted for the majority of significant findings (80%; 20/25). Reverse MR analysis did not support reverse causality. Sensitivity analyses confirmed the robustness of these associations, with no significant horizontal pleiotropy or heterogeneity detected. Our study suggests potential causal associations between blood levels of glutamine, citric acid, glucose, various lipids, and lipoproteins with AA. Initial analyses found no evidence of reverse causality. These findings require further investigation for validation.

## 1. Introduction

Alopecia areata (AA) is a hair loss disorder resulting from an autoimmune response. It is characterized by patchy hair loss on the scalp, which can progress to complete baldness on the scalp and body. This condition affects approximately 2% of the global population and detrimentally affects individual quality of life. The prevailing hypothesis concerning the pathogenesis of AA posits that it is initiated by the loss of immune privilege in hair follicle (HF), leading to a T cell-mediated attack.^[1,2]^ AA demonstrates no marked ethnic or gender predominance and may arise at any age, although onset is most common before the age of 40. A significant proportion of patients report experiencing social stigma and emotional distress, with elevated rates of anxiety and depressive disorders observed in this population. Furthermore, AA is frequently comorbid with other autoimmune conditions, including thyroid disease, atopic dermatitis, and vitiligo.^[3]^

Recent advances in metabolomics have greatly expanded our understanding of disease mechanisms, therapeutic development, and precision medicine approaches in complex disorders. It is estimated that genetic factors contribute to roughly 50% of the variability in metabolite levels, underscoring the potential of genetic instruments to infer causal relationships between metabolites and disease phenotypes.^[4]^ Accumulating studies have implicated circulating metabolites in the pathogenesis of several inflammatory skin diseases, such as psoriasis, atopic dermatitis, and vitiligo. In particular, elevated β-hydroxybutyrate has been shown to potentiate inflammatory immune activity in AA, correlating with chronic, refractory hair loss.^[5–7]^ Serum β-hydroxybutyrate may thus represent a promising prognostic biomarker in severe AA.

However, due to ethical constraints and the progressive nature of AA, interventional studies such as randomized controlled trials remain challenging to conduct. Mendelian randomization (MR) has emerged as a powerful epidemiological tool for strengthening causal inference by employing genetic variants (typically single-nucleotide polymorphisms [SNPs]) as instrumental variables (IVs).^[8]^ This approach mitigates limitations inherent in conventional observational designs, such as reverse causation and unmeasured confounding, thereby providing more robust evidence regarding exposure–outcome relationships. In this study, we leverage a 2-sample MR framework to evaluate the putative causal effects of blood metabolites on AA, offering novel insights into the metabolic underpinnings of this complex autoimmune disorder.

## 2. Materials and methods

**Figure 1.**
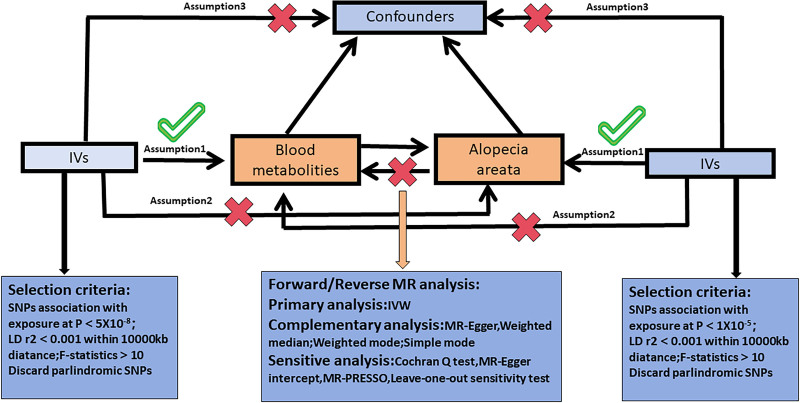
Overview of this MR analysis. MR = Mendelian randomization.

This study employed a 2-sample MR approach to investigate the causal relationship between blood metabolites and AA. Using the inverse variance weighted (IVW) method, we performed a forward MR analysis to identify potential metabolites associated with the risk of AA. In addition, a reverse MR analysis was performed to assess the effect of AA on the blood metabolites that were identified (Fig. 1).

MR relies on 3 fundamental assumptions: IVs must be strongly associated with the exposure (blood metabolites). IVs should affect AA solely through blood metabolites, exhibiting no pleiotropic effects. IVs must not be influenced by any confounders between blood metabolites and AA. Adherence to these assumptions is crucial to validate the MR-derived causal inferences between the exposure and outcomes. There was no need for ethical approval or informed consent in this study since it relied on publicly available, summary-level genome-wide association study (GWAS) data. Additionally, all original studies had already obtained the necessary approvals.^[9]^

### 2.1. GWAS data source

To reduce population stratification, analyses were restricted to individuals of European ancestry. Blood metabolite data were obtained from a large-scale GWAS meta-analysis of 123 circulating metabolites (including lipoprotein subclasses, amino acids, fatty acids, and glycolysis-related metabolites) quantified via high-throughput NMR metabolomics.^[10]^ The study included 24,925 Europeans from 33 cohorts, with values adjusted for age, sex, and fasting status. Data are available via the OpenGWAS platform. AA data were sourced from the FinnGen project, including 682 AA cases and 361,140 Finnish controls. Summary statistics are accessible on the FinnGen website under the phenotype code: finn-b-L12_ALOPECAREATA. All data are publicly available, and each original study obtained ethical approval.

### 2.2. Selection of IVs

In this MR study, forward MR employed a stringent instrument selection threshold (*P* < 5 × 10⁻⁸), while reverse MR used a relaxed threshold (*P* < 1 × 10⁻⁵) to secure sufficient SNPs for sensitivity analyses given the limited genome-wide significant variants in disease GWAS.^[11]^ We performed linkage disequilibrium clumping with an *r*² < 0.001 and a distance window of 10,000 kb to ensure SNP independence. Instrument strength was evaluated via the *F*-statistic, calculated as *F* = [*R*² × (N − 2)]/(1 − *R*²), and only SNPs with *F* > 10 were retained to avoid weak instrument bias.^[12]^

### 2.3. Statistical analysis

Using R version 4.3.1 (The R Foundation for Statistical Computing, Vienna, Austria) and the 2-sample MR package, an MR analysis was conducted. The IVW approach was mainly used to evaluate the association between AA and blood metabolites, with a significance level of *P* < .05. To make causal inference more accurate and reliable, further analyses were conducted. Some of these methods were MR-Egger regression, weighted median method, and the weighted mode. The IVW approach is deemed to have produced a favorable result when other methods’ beta values in the same direction do not exhibit pleiotropy or heterogeneity.^[13]^

The Cochran *Q* test was used with both MR-Egger and IVW methods, and a *P*-value >.05 suggests no heterogeneity among the IVs. The MR-Egger intercept test is designed to detect horizontal pleiotropy among IVs and test for violations of the 2nd MR assumption. The lack of pleiotropy is indicated by a *P*-value higher than .05. After identifying and removing outlier results using the MR-PRESSO method, a reanalysis was conducted. The value .05 was chosen as the significance level α. An evaluation was conducted to assess the impact of individual SNPs on MR results using a “leave-one-out sensitivity test.” Consistency in outcomes following the elimination of any SNP demonstrates the resilience of the results.^[14,15]^ The results of the study were given as odds ratio (OR) with the associated 95% confidence interval (CI). A substantial causal association was defined as a *P*-value < .05. This analysis is conducted using publicly available summary data and does not require ethical approval.

## 3. Results

**Figure 2.**
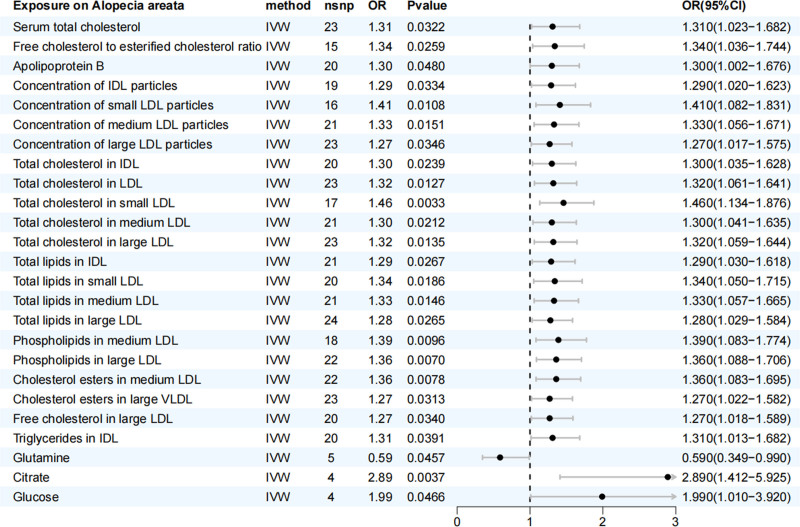
MR analysis of the causal effects of blood metabolites on AA. Forest plot showing the ORs and 95% CIs for the associations between 25 metabolites and AA risk, generated using the IVW method. An OR > 1 indicates a potential risk factor, while an OR < 1 suggests a potential protective factor. The vertical line at OR = 1 represents the null effect. AA = alopecia areata, CI = confidence interval, IVW = inverse variance weighted, MR = Mendelian randomization, OR = odds ratio.

Following the research criteria, 123 circulating metabolites were screened, yielding 1376 SNPs as IVs. All SNPs displayed *F*-statistics >10, with a minimum *F*-statistic of 30.5, indicating suitability for MR analysis (Table S1, Supplemental Digital Content, https://links.lww.com/MD/Q890). Using the IVW model, we assessed the causal relationship between these metabolites and AA. The MR-Egger regression, WM, and weighted mode consistently showed the same directions for the β-values. This analysis identified 25 metabolite traits significantly associated with AA (*P* < .05); the IVW forest plots for these metabolites are shown in Figure 2.

### 3.1. Effects of metabolites on AA

Based on the systematic analysis of 25 metabolites significantly associated with AA, the results reveal distinct class-specific patterns, with lipoprotein-related metabolites constituting the majority (n = 20, 80%). The findings are summarized below by metabolic category (Fig. 2). Lipoprotein and lipid metabolites formed the most prominent group. Among these, several subclasses showed significant positive associations with AA. General cholesterol-related measures, including serum total cholesterol (OR = 1.31, 95% CI = 1.02–1.68) and the ratio of free cholesterol to esterified cholesterol (OR = 1.34, 95% CI = 1.04–1.74), were elevated. Apolipoprotein B also exhibited a positive association (OR = 1.30, 95% CI = 1.00–1.68). The concentrations of various lipoprotein particles were significantly increased, specifically intermediate-density lipoprotein (IDL) (OR = 1.29), small low-density lipoprotein (LDL) (OR = 1.41), medium LDL (OR = 1.33), and large LDL particles (OR = 1.27). Furthermore, the total cholesterol content within specific lipoprotein fractions was higher, including in IDL (OR = 1.30), LDL (OR = 1.32), small LDL (OR = 1.46), medium LDL (OR = 1.30), and large LDL (OR = 1.32). The analysis of lipid components also revealed positive associations for total lipids across IDL (OR = 1.29), small LDL (OR = 1.34), medium LDL (OR = 1.33), and large LDL (OR = 1.28), as well as for phospholipids in medium LDL (OR = 1.39) and large LDL (OR = 1.36). Additional significant lipid metabolites included cholesterol esters in medium LDL and large very low-density lipoprotein, and triglycerides in IDL. Outside the lipid domain, 3 other metabolite categories were identified. The amino acid metabolite, glutamine, was uniquely and inversely associated with AA (OR = 0.59, 95% CI = 0.35–0.99). Among organic acids and carbohydrates, both citrate (OR = 2.89, 95% CI = 1.41–5.92) and glucose (OR = 1.99, 95% CI = 1.01–3.92) showed strong positive associations. In conclusion, the metabolomic profile of AA is overwhelmingly characterized by perturbations in lipoprotein and lipid metabolism, with additional involvement of energy metabolism substrates (citrate and glucose). The sole protective association observed with glutamine highlights a potential role for amino acid metabolism in the disease.

#### 3.1.1. Pleiotropy and sensitivity analysis

A sensitivity analysis was carried out to enhance the reliability of the results. No heterogeneity among the IVs was identified after data analysis utilizing the Cochran *Q* test, MR-Egger, and IVW techniques (*P* > .05). Further evidence that no horizontal pleiotropy exists (*P* > .05) was provided by the MR-Egger regression intercept (Table 1, Tables S2 and S3, Supplemental Digital Content, https://links.lww.com/MD/Q890). The influence of a single SNP, as determined by the leave-one-out method, had a minimal effect on the causality of the 25 metabolite signatures.

**Table 1:** Results of the Cochran *Q* test and MR-Egger regression. The Cochran *Q* test, utilizing MR-Egger and IVW, revealed no heterogeneity among IVs (*P* > .05). MR-Egger regression intercept results also indicated no horizontal pleiotropy (*P* > .05).

Heterogeneity			Pleiotropy		
Exposure	MR Egger Q _ p	IVWQ _ p	Egger intercept	Egger *P*	MR-Presso *P*
Serum total cholesterol	0.975	0.952	-0.038	.174	.009
Free cholesterol to esterified cholesterol ratio	0.999	0.999	-0.018	.599	0
Apolipoprotein B	0.997	0.991	-0.041	.221	.005
Concentration of IDL particles	0.95	0.937	-0.035	.29	.01
Concentration of small LDL particles	0.935	0.937	-0.03	.423	.003
Concentration of medium LDL particles	0.981	0.966	-0.037	.222	.003
Concentration of large LDL particles	0.958	0.917	-0.039	.142	.013
Total cholesterol in IDL	0.738	0.775	-0.015	.595	.017
Total cholesterol in LDL	0.854	0.857	-0.023	.386	.007
Total cholesterol in small LDL	0.977	0.986	-0.002	.949	0
Total cholesterol in medium LDL	0.942	0.902	-0.039	.173	.008
Total cholesterol in large LDL	0.98	0.983	-0.017	.515	.002
Total lipids in IDL	0.839	0.858	-0.018	.513	.014
Total lipids in small LDL	0.957	0.934	-0.039	.227	.005
Total lipids in medium LDL	0.98	0.967	-0.035	.234	.003
Total lipids in large LDL	0.938	0.916	-0.03	.23	.01
Phospholipids in medium LDL	0.834	0.837	-0.03	.39	.006
Phospholipids in large LDL	0.952	0.957	-0.02	.488	.001
Cholesterol esters in medium LDL	0.993	0.994	-0.018	.53	0
Cholesterol esters in large VLDL	0.969	0.945	-0.035	.187	.009
Free cholesterol in large LDL	0.948	0.922	-0.033	.225	.012
Triglycerides in IDL	0.998	0.999	-0.017	.669	.001
Glutamine	0.952	0.553	0.097	.2	.083
Citrate	0.656	0.728	-0.135	.567	.022
Glucose	0.834	0.923	0.042	.766	.016

IDL = intermediate-density lipoprotein, IVs = instrumental variables, IVW = inverse variance weighted, LDL = low-density lipoprotein, MR = Mendelian randomization.

### 3.2. Effects of AA on blood metabolites

**Figure 3.**
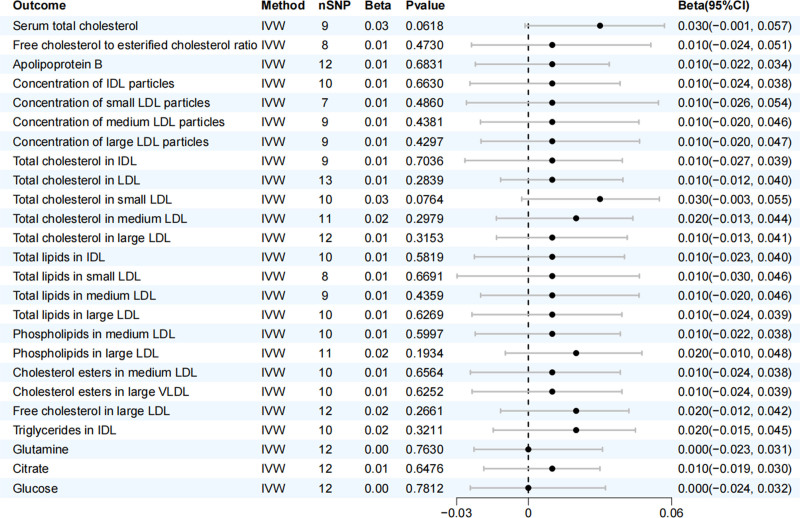
Forest plot of the reverse MR analysis evaluating the causal effects of AA on 25 blood metabolites. Each line represents the beta coefficient (β) and 95% CI for a specific metabolite. A β > 0 suggests that genetic predisposition to AA is associated with an increase in the metabolite level, while a β < 0 suggests an association with a decrease. The vertical line at β = 0 represents the null effect. AA = alopecia areata, CI = confidence interval, MR = Mendelian randomization.

For the purpose of investigating potential reverse causal effects, a reverse MR analysis was conducted. The exposure in this analysis was AA, while the outcomes were the 123 blood metabolites. When the number of SNPs linked to the exposure is limited and does not meet the strict genome-wide significance levels, researchers often use a lower significance threshold (e.g., 1.00 × 10^-5^) to identify broader differences. This approach significantly boosts the statistical power in this particular context.^[16]^ Reverse MR identified significant gene loci related to AA (*P* < 1 × 10^-5^), with other methods aligning with those used in forward MR. Results indicated no causal relationship between AA and the metabolites (Fig. 3).

## 4. Discussion

The causal effects of blood glutamine, citric acid, and glucose on AA remain underexplored, despite known impacts on HF. Glutamine serves as a critical energy source for HFs, with its metabolism regulating HF stem cell fate reversibility and promoting regeneration signaling.^[17]^ Specifically, the mTORC2–Akt signaling pathway governs glutamine metabolism, which is essential for HF stem cell regeneration and maintenance.^[18]^ Diminished glutamine may disrupt this process, impairing HF regeneration and contributing to AA pathogenesis: consistent with the protective association observed in our study. As a key TCA cycle intermediate, citric acid is vital for cellular energy production. However, TCA cycle disruption, particularly citrate accumulation, has been shown to inhibit hair growth. Experimental evidence indicates that knockdown of citrate synthase, which reduces citric acid production, can reverse hair growth inhibition and alleviate inflammation and apoptosis in follicular cells.^[19]^ Elevated blood citrate may therefore reflect or induce a dysfunctional metabolic state in HF, potentially triggering local inflammation and apoptosis, thereby contributing to AA development.

Clinical and mechanistic evidence suggests a significant association between AA and type 2 diabetes mellitus, with dysregulated glucose metabolism potentially serving as a key link. A large-scale case–control study indicated a significantly higher prevalence of prediabetes in AA patients compared to healthy controls (OR = 1.62).^[20]^ Mechanistically, animal studies have demonstrated that the hyperglycemic environment characteristic of diabetes can suppress the canonical Wnt/β-catenin signaling pathway, which is crucial for HF stem cell activation and regeneration.^[21]^ This impairment may contribute to dysfunctional HF regeneration, thereby promoting AA pathogenesis. Thus, glucose metabolic disturbances may represent an important shared pathophysiological basis connecting AA and T2DM. Our study extends recent MR evidence on metabolites and AA.^[22]^ In contrast to the research by Lei et al, which primarily identified specific metabolite molecules as risk factors, our analysis provides a more comprehensive examination of lipoprotein subfractions, encompassing particle density, cholesterol, and phospholipid content within IDL and LDL subclasses. Crucially, we uniquely identified glutamine as a novel protective factor and citrate as a potential risk factor: thereby revealing a broader spectrum of metabolic disturbances in individuals with AA.

Our MR analysis provides genetic evidence supporting a causal role of specific lipoprotein subfractions (particularly IDL and LDL particles) in the pathogenesis of AA, which aligns with prior findings by Chen et al.^[23]^ Specifically, IDL components (total lipids, cholesterol, triglycerides) and LDL subfractions (particle concentrations and compositional lipids) were positively associated with AA, indicating that an atherogenic dyslipidemia profile contributes significantly to AA risk. Potential mechanisms may involve immunometabolic pathways. Disruption of cholesterol homeostasis could impair follicular blood supply via endothelial dysfunction, while lipid anomalies might amplify inflammatory processes through shared immune pathways.^[24]^ This is supported by reported causal links between plasma lipids and atopic dermatitis, as well as associations between serum lipids and asthma, suggesting comorbidity mechanisms between AA and immune-inflammatory diseases.^[25,26]^ Further evidence comes from clinical correlations between AA and metabolic syndrome, and novel biomarkers such as Lipocalin-2 and insulin have been proposed to reflect AA-related metabolic dysregulation.^[27,28]^ Clinically, lipid-modulating drugs may offer therapeutic benefits, as demonstrated by simvastatin/ezetimibe treatment promoting hair regrowth in AA patients.^[29]^ In summary, our MR study clarifies the causal contributions of lipoprotein subfractions to AA and highlights lipid metabolism as a potential target for metabolic intervention strategies.

Several limitations warrant consideration. First, analyses were restricted to individuals of European ancestry. This enhances internal validity but limits generalizability; replication in non-European populations is needed to assess transferability. Second, minor sample overlap (particularly involving Finnish cohorts) cannot be fully excluded. While any bias in 2-sample MR estimates is likely modest given the large imbalance in contributing sample sizes, we cannot rule it out. Third, in the MR screen of 123 metabolites, 25 associations were nominally significant (*P* < .05) before multiple-testing correction; none met a conventional FDR threshold (FDR < 0.05). Applying a more exploratory threshold (FDR < 0.20) identified suggestive signals (Table S4, Supplemental Digital Content, https://links.lww.com/MD/Q890). Although sensitivity analyses (MR-Egger, MR-PRESSO, leave-one-out) did not indicate substantial pleiotropy or instability, all associations should be considered preliminary and require confirmation in independent cohorts with more stringent validation. Given the exploratory scope, these results are best viewed as hypothesis-generating. Future work should prioritize external replication across diverse ancestries and incorporate mechanistic studies to elucidate underlying biological pathways.

## 5. Conclusion

This study employs a bidirectional MR approach to investigate the potential causal relationships between blood metabolites and AA. Our analyses revealed suggestive associations of AA with blood levels of glutamine, citrate, glucose, and specific lipid and lipoprotein fractions. No robust evidence for reverse causality was detected. These findings should be interpreted as hypothesis-generating, underscoring the need for validation in independent cohorts and further investigation into the underlying biological mechanisms.

## Acknowledgments

The authors extend their gratitude to all participants and researchers for their valuable contributions to the GWAS data submissions.

## Author contributions

**Methodology:** Xiao-Shuang Yang, Ru-Dai, Sheng-Zhao Chen.

**Resources:** Xiao-Shuang Yang.

**Supervision:** Ru-Dai, Zhong-Fa Lv.

**Writing – original draft:** Xiao-Shuang Yang, Yu-Jie Miao.

**Writing – review & editing:** Ru-Dai, Zhong-Fa Lv.

## Supplementary Material
